# Editorial: Petroleum Microbial Biotechnology: Challenges and Prospects

**DOI:** 10.3389/fmicb.2017.00833

**Published:** 2017-05-12

**Authors:** Wael A. Ismail, Jonathan D. Van Hamme, John J. Kilbane, Ji-Dong Gu

**Affiliations:** ^1^Environmental Biotechnology Program, Life Sciences Department, College of Graduate Studies, Arabian Gulf UniversityManama, Bahrain; ^2^Department of Biological Sciences, Thompson Rivers UniversityKamloops, BC, Canada; ^3^Department of Biology, Illinois Institute of TechnologyChicago, IL, USA; ^4^School of Biological Sciences, University of Hong KongHong Kong, Hong Kong

**Keywords:** hydrocarbon, oil reservoir, corrosion, enhanced oil recovery, methanogenesis, biosurfactants, biodesulfurization, energy

It has become evident that fossil fuels such as petroleum will continue to contribute a major fraction of the energy portfolio worldwide for the coming decades. The United States Energy Information Administration anticipates a growth of global oil demand up to 123 million barrels per day by 2025 (Sahu et al., 2015). Accordingly, oil production and processing operations are expanding continuously to meet the accelerated growth in global energy demand.

Unfortunately, serious environmental pollution issues are associated with petroleum recovery, transportation, and refining. In addition, these processes are energy-intensive, costly, and, in some cases, not sufficiently efficient (Gray, 1994; Kilbane, 2006; Ramirez-Corredores and Borole, 2006). Furthermore, the increased demand for fossil fuels will inevitably force the oil industry to produce and refine increasing amounts of unconventional resources such as heavy and extra-heavy crudes as well as bitumen. This will lead to even more environmental issues in addition to technical challenges for the oil industry (Ramirez-Corredores and Borole, 2006; Speight, 2013).

The continuously rising global demand for cleaner fuels, together with the depletion of light crude oil resources and strict environmental regulations, have provoked the need for alternative or complementary novel technologies for oil production and refining. The interaction between microorganisms and petroleum hydrocarbons has been well recognized and the intimate contact between them starts in oil-bearing subsurface formations (Ehrlich et al., 2016). This constituted the basis from which petroleum biotechnology has emerged. Petroleum biotechnology exploits the astonishing metabolic and adaptive capabilities of dedicated hydrocarbon-degrading/transforming microorganisms (Van Hamme et al., 2003; Mbadinga et al., 2011). As compared to conventional thermochemical and physical approaches, biotechnology-based processes are generally environmentally friendly, economic, and are characterized by high selectivity (Le Borgne and Quintero, 2003; Kilbane, 2006).

Petroleum biotechnology has been applied for environmental cleanup of oil spills and biological treatment of refinery wastes (bioremediation). Other emerging applications include oil exploration, microbial enhanced oil recovery (MEOR), biodesulfurization and biodenitrogenation of distillates, biodemetallation, bioupgrading of heavy crudes and refining residues, valorization of refining wastes, bioconversion of residual oil to methane, control of oil field souring and corrosion, formulation of petrochemicals, etc. (Vazquez-Duhalt and Quintero-Ramierez, 2004; Morales et al., 2010; Mbadinga et al., 2012; Wang et al., 2012, 2014; Zhou et al., 2012, 2016; Bachmann et al., 2014; Bian et al., 2015; Head and Gray, 2016).

With the exception of bioremediation and enhanced oil recovery, most of these applications are still in the research and development phase in laboratories (Morales et al., 2010; Bachmann et al., 2014). For commercial application, intensive research and development work is still needed to thoroughly understand the structure, function and ecophysiology of microbial communities inhabiting oilfields, refineries as well as oil- and hydrocarbon-impacted ecosystems. This direction of research has been greatly advanced by both culture-independent and conventional enrichment approaches. Currently, the power of metagenomics afforded by rapidly evolving advanced sequencing techniques can be harnessed for comprehensive structural and functional characterization of microbial communities to advance knowledge on the active microbial community members and the functional genes expressed in subsurface ecosystems and other environments (Joshi et al., 2014; Tan et al., 2015).

A major challenge that impedes the development of further biotechnological applications for the petroleum industry is the complex, heterogeneous, and hazardous nature of crude oil and various distillates, residues and wastes. Successful application of petroleum biotechnology is based on the development of robust microbial biocatalysts that can adapt to and tolerate the various hydrocarbon substrates, and perform the desired biodegradation or biotransformation reactions at commercially viable rates. Recent advances in the field of hydrocarbon activation under anoxic conditions have enriched the scientific knowledge on new biochemical processes, and the fumarate-addition pathway is an example as evidenced in enrichment cultures and oilfields (Mbadinga et al., 2011; Bian et al., 2015; Zhou et al., 2016). In this context, microbial consortia, naturally occurring or reconstituted, offer many advantages as compared to axenic strains for energy recovery or production of value-added intermediate chemicals (McGenity et al., 2012; Mikesková et al., 2012).

In this research topic, we received 19 articles that cover 6 applications on different aspects of petroleum biotechnology. Three papers focus on microbial enhanced oil recovery, 3 papers address methanogenic degradation of hydrocarbons and CO_2_ sequestration, 3 papers investigate bioremediation, 4 articles characterize microbial communities, 4 articles discuss microbiologically influenced corrosion, 1 article addresses biodesulfurization, and 1 is an opinion article. In the following, we briefly describe these articles in chronological order starting with the most recent.

Dong et al. investigated microbial enhanced oil recovery using rhamnolipid biosurfactants produced by a novel strain of *Acinetobacter junii*. They show significant increase in oil recovery due to reduction of interfacial tension, alteration of wettability and mobility of microorganisms. Liang et al. performed functional and structural characterization of alkane-degrading methanogenic enrichment cultures, employing long-term incubation to eliminate inactive members and accumulate the key players. They also analyzed the degradation process and the functional genes involved. Nie et al. highlight factors that shape taxonomic and functional composition of microbial communities in oil reservoirs using shotgun sequencing of metagenomes from geographically distant oil reservoirs.

Obi et al. adopted Illumina sequencing of 16S rRNA genes to explore the impact of pollutant type and level on microbial community structure and function in estuarine sediments of Lagos lagoon (Nigeria) to identify key hydrocarbon degraders and those affected by the pollution. In a study on microbially influenced corrosion, Liang et al. establish the role of the halophilic *Halanaerobium* as a corrosion-causing bacterium in a gas production field. They also report that the dominant *Halanaerobium* strain degrades guar gum, which is used in fracture fluids to produce acetate and sulfide. The impact of substrate complexity on the community structure of methanogenic hydrocarbon-degrading enrichment is described by Fowler et al. Their findings highlight the stability of syntrophic interactions regardless of substrate diversity and show the importance of syntrophic interactions amongst the members of complex methanogenic communities.

In another work on corrosion, Voordouw et al. used uniformly sized carbon steel beads for monitoring corrosion in oil fields. For bioremediation of desert soil, Al-Kindi and Abed applied biostimulation to oil-contaminated desert soil with sewage sludge, wheat straw, and soybean meal. They presented data on oil degradation and shifts in microbial community composition. The biodesulfurization potential of mixed cultures is addressed by Ismail et al., with the authors showing how the sulfur source affects the structure of a biodesulfurizing mixed culture. They also studied the biodesulfurization spectrum and catalytic activity. Gao et al. shed light on stimulation of microbial enhanced oil recovery using a lipopeptide-producing *Bacillus subtilis* strain that is added during water flooding of oil reservoirs.

Aüllo et al. describe two *Desulfotomaculum* populations as key degraders of monoaromatic compounds in a deep subterranean gas storage aquifer. They also present carbon and hydrogen isotopic fractionation data that highlight the mechanism of anaerobic benzene activation under sulfate-reducing conditions. In his opinion article, Kilbane reviews the potential contributions of biotechnology to the future energy industry and discusses the underlying challenges. Muhr et al. developed a fluorescent bioreporter for acetophenone using the acetophenone catabolic operon as a scaffold. They show that the mCherry-based bioreporter is specific to acetophenone and two enantiomers of 1-phenylethanol, which are transformed to acetophenone and has potential application in environmental monitoring and petroleum exploration.

An et al. applied metagenomics to analyze sodium bisulfite impact on corrosion-provoking microbial communities in bitumen production pipelines. They show increased abundance of hydrogen-utilizing delta and epsilonproteobacteria after excess application of sodium bisulfite as an oxygen scavenger. Mand et al. investigated how methanogens and acetogens play a role in microbial influenced corrosion in low-temperature oil reservoirs using carbon steel organics as an electron donor. Xue and Voordouw studied the combination of biocides and nitrate for control of oil reservoir souring. Elshafie et al. characterized sophorolipid biosurfactants produced by *Candida bombicola* and explored their applicability for microbial enhanced oil recovery.

Ren et al. draw our attention to the impact of allochthonous bacteria, introduced into oil reservoirs during water flooding, on indigenous microbial communities. They present permeability of the strata and reservoir conditions as determinants for the abundance of the bacteria injected in the production well. Liu et al. performed functional characterization of microbial communities in CO_2_-flooded oil reservoirs and highlight the potential conversion of CO_2_ into methane using functional genes of methanogens and hydrogenases as biomarkers.

The collection of research results compiled in this research topic is by no means exhaustive. It merely serves to showcase current understanding of some petroleum biotechnology applications from active researchers around the world. Though very challenging, the potential of biotechnology in the fossil fuel industry is immense and the coming decades will witness considerable progress in the field. This will be fueled by advances in relevant disciplines such as metabolic engineering, bioprocess technology, biochemical engineering, as well as biocatalysis. In particular, the field of artificial metalloenzymes has turned into a vibrant research area, which can be exploited for development of novel hydrocarbon biotransformations. New knowledge on hydrocarbon biodegradation and biotransformation has been derived from recent research, especially from studies on specific biochemical processes and the underlying genes and microorganisms. Recent metagenomics techniques coupled with stable isotope probing, single cell sequencing, metaproteomics and metabolomics will illuminate new insights into microbial ecophysiology in subsurface ecosystems. At the same time, synthetic biology will play an important role in the optimization of the assembly of selected functions into engineered organisms or consortia to realize purpose-directed development of efficient biocatalysts. In parallel with scientific advances, extensive collaboration between academia and the fossil fuel industry is essential to ensure successful development and implementation of biotechnology applications that tackle specific technical, economic, and environmental issues. Overall, while petroleum has deep roots in the history of microbiology, it is clear that the subject remains critically important for industry and the environment and will remain an active area of fundamental and applied research for decades to come.

## Author contributions

All authors listed, have made substantial, direct and intellectual contribution to the work, and approved it for publication.

### Conflict of interest statement

The authors declare that the research was conducted in the absence of any commercial or financial relationships that could be construed as a potential conflict of interest.
